# The Cover Page

**Published:** 1918-12

**Authors:** 


### The Cover Page.

Largely through the courtesy of Sharp & Dohme, who have an annual contract for the lower half of the page, the cover page of this issue of the Texas Medical Journal is given over to the Red Cross, in recognition of its wonderful services rendered during the war. The work done by this organization in this war has been simply marvelous and the people the world over are anxious to pay tribute to it. Whenever and wherever the Red Cross makes a call for help in future every person who knows anything of what it has done to alleviate suffering will respond to the appeal, and at this Christmas time it is particularly appropriate that those who would assist in healing should make contributions to that organization that gives its entire energies to relieving suffering and distress. Put your name to the Red Cross Christmas Roll Call.

